# Pasture-based fattening does not cause severe nonperforating lesions in veal calves of dairy breeds

**DOI:** 10.3168/jdsc.2022-0370

**Published:** 2023-10-03

**Authors:** Georg Simon, Edna Hillmann, Kerstin Barth

**Affiliations:** 1 Johann Heinrich von Thünen Institute, Federal Research Institute for Rural Areas, Forestry and Fisheries, Institute of Organic Farming, Trenthorst 32, 23847 Westerau, Germany; 2 Humboldt-Universität zu Berlin, Faculty of Life Sciences, Albrecht Daniel Thaer-Institute of Agricultural and Horticultural Sciences, Philippstraße 13, 10115 Berlin, Germany

## Abstract

Abomasal damage is a known health issue in intensive veal production and is associated with improper housing and feeding. Grass-based veal production could be an improvement, as access to pasture allows the expression of natural behaviors, such as species-specific foraging, and thus can contribute to calf welfare. However, data on the abomasal health of grazed calves have not yet been reported. As feed composition can affect the severity of mucosal damage, we compared the abomasa of calves that grazed on 2 swards with different plant composition. From 2018 to 2020, 111 calves of 2 dairy breeds, German Holstein and Jersey, were kept in whole-day strip grazing on these swards. Calves were sampled regularly for intestinal parasites and anthelmintic drug use was recorded. The animals were slaughtered at an age of 32 ± 2 wk (mean ± standard deviation) after individually varying grazing periods of 75 ± 22 d. After slaughter, dissection revealed that 104 of the 111 abomasa displayed low-grade nonperforating lesions, which were primarily located in the pyloric part. A more severe nonperforating lesion was registered in one calf only. In 46 abomasa, we observed diffuse inflammation. This inflammation was characterized by normal mucosal rugae but with slight discoloration that varied in the degree of redness and proliferation of the mucosa. Sward composition and breed did not affect the occurrence of abomasal lesions, but treatment with anthelmintics was negatively correlated with diffuse inflammation. Compared with the abomasa of calves fed under intensive conditions, the abomasa of calves kept on pasture in this study were not as severely altered, but completely unaffected abomasa were rarely observed either. This raises the question to what extent the observed changes in the abomasum were caused by husbandry and feeding conditions or whether they correspond to the normal condition of grazing animals.

## Highlights

• Grazing did not cause severe lesions, in abomasa of dairy bull calves.
• Besides nonperforating lesions, diffuse inflammations were observed.
• An herb-enriched grass-clover sward did not affect the occurrence of abomasal lesions.
• More lesions were found in the pylorus than in the fundus.

## Graphical Abstract

**Figure:**
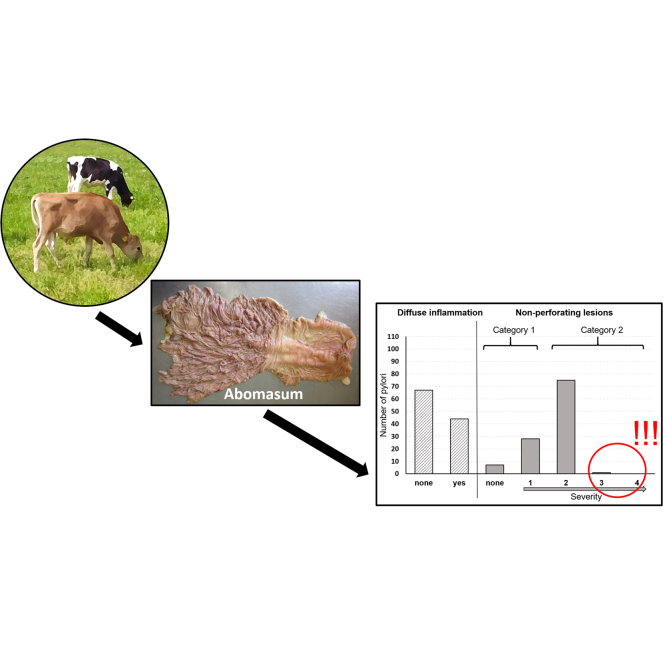
**Summary:** Abomasal lesions are associated with inadequate housing and feeding, and have been found in intensively fattened veal calves. Fattening on pasture might improve abomasal health or even prevent abomasal lesions in calves. This was confirmed by the absence of severe nonperforating lesions in 111 abomasa of grazed bull calves. However, less severe lesions were found in 104 of these abomasa, of which 44 showed diffuse inflammation that had never been described before. This inflammation was characterized by normal mucosal rugae but with slight discoloration that varied in the degree of redness and proliferation. However, these mild lesions and inflammation raise the question of the extent to which the observed changes in abomasa were caused by housing and feeding conditions, or whether they somewhat corresponded to the normal condition of grazing animals.

The overall prevalence of nonperforating abomasal lesions at slaughter in Europe ranges from 67% to 95% in veal calves (reviewed by Bähler et al., 2010). These lesions are considered multifactorial damages that may indicate deficiencies in husbandry, nutrition, and management (reviewed by Bus et al., 2019). The pathogenesis of abomasal lesions is a stress-induced disturbance of the balance between protective and aggressive mechanisms affecting the mucosa (reviewed by Hund and Wittek, 2017).

Nonperforating abomasal lesions are erosive mucosal defects that can heal via epithelial regeneration without scar formation. However, they can also exacerbate deep hemorrhagic craters and result in perforating ulcers (reviewed by Smith et al., 1983). It is suspected that exacerbation is caused by the enabled diffusion of hydrogen ions back from the lumen into the mucous membrane and the penetration of the proteolytic enzyme pepsin into deeper layers of the abomasal wall, possibly resulting in peritonitis, anemia, or, ultimately, death (reviewed by Braun et al., 2019). Further clinical symptoms may include anorexia and abdominal pain as well as dehydration (Palmer and Whitlock, 1984). However, visible external symptoms often appear nonspecific and inconspicuous in living individuals, complicating the diagnosis ante mortem (Munch et al., 2019). Hence, affected animals often remain undetected (reviewed by Hund and Wittek, 2017).

To obtain light pink meat, veal calves have traditionally been fed large amounts of milk replacer and limited amounts of solid feed, although a higher proportion of roughage is physiologically necessary for rumen development (Brscic et al., 2011). Although the amount of solid feed has increased substantially, the amount of roughage feed remains insufficient (reviewed by Webb et al., 2013). In relation to this diet, nonperforating abomasal lesions and perforating ulcers were commonly observed in calves. However, the effects of diet are not yet fully understood (reviewed by Bus et al., 2019) as feeding large amounts of crude fiber can also mechanically damage the abomasum (Webb et al., 2013).

On the other hand, grazing could have a positive effect on stomach health. Plant secondary metabolites, as found in swards with large proportions of herbs, can have antiseptic effects (Athanasiadou and Kyriazakis, 2004), and anthelmintic substances have been detected in some herbs (Peña-Espinoza et al., 2017). Furthermore, access to pasture allows the expression of natural behaviors, such as species-specific foraging. Thus, grass-based veal production could be an improvement not only for feeding but also for housing. However, there is little information on how grazing itself and sward composition affect abomasal health of calves. Therefore, we hypothesized that grazing would reduce the risk of nonperforating abomasal lesions in veal calves of dairy breeds, and that pastures enriched with herbs would cause even less abomasal damage than pure pastures with ryegrass and clover.

The study was conducted in the grazing seasons of 2018 to 2020 at the research farm of the Thünen Institute in Northern Germany (53°46′8.335″N, 10°31′10.531″E), animal experiment number V244–29138/2019, MELUND Schleswig-Holstein according to the German Animal Welfare Act (Federal Republic of Germany, 2018). Farm management followed European Union Regulation No. 889 (EC, 2008) for organic production.

The minimum sample size was determined using a 2-sample *t*-test power calculation: n = 22 calves for each of 2 treatments, d = 100 g of daily weight gain, s = 131.35, α = 0.05, β = 0.2, and power = 0.80.

Of the 111 bull calves included in this study, 39 were born at the research farm. The remaining 72 animals were purchased at an age of 27 ± 8 d from surrounding organic farms. In 2018, 2019, and 2020, the number of calves varied due to different availability: 18, 29, and 33 German Holstein and 10, 8, and 13 Jersey calves, respectively.

From the 39 calves born at the research farm, 28 were kept with their dam for 5 to 7 d in the calving pen and were afterward reared in a whole-day full contact system (Sirovnik et al., 2020) for 93 ± 3 d. The other 11 calves were separated from the dam after birth and kept in single calf hutches for 7 d. Thereafter, 6 calves were group-housed in a calf creep with an outdoor run (at least 2.1 and 2.9 m^2^·animal^−1^ inside and outside, respectively), and warm whole milk was provided using an automatic feeder (Förster-Technik GmbH). All other calves were put into groups of up to 12 animals in calf igloos (at least 1.2 m^2^·animal^−1^) with a veranda (at least 2.1 m^2^·animal^−1^) and fed acidified cold whole milk with a bucket feeder with teats (Patura KG). Over 3 mo a quantity of 1,100 L·calf^−1^ was offered. Milk intake of calves with dam contact could not be measured but should have a similar amount based on Barth (2020). Each animal was provided with concentrate pellets (1,500 g·d^−1^) and ad libitum access to water, hay, and a mixed ration of 63.5% grass silage, 30.2% corn silage, 5.2% concentrates, and 1.2% minerals over the entire rearing period, until grazing on the experimental plot started. Straw was used as bedding for all housings.

Grazing with the dam began at an age of 55 ± 23 d. Before the separated or purchased calves entered the experimental plots, they had their first access to pasture (paddock size: 400 m^2^) in direct proximity to their accustomed housings at the age of 98 ± 16 d. While this period lasted 2 d in 2018 and 2019, it was extended to 2 wk in 2020. After early grazing, the weaned calves were evenly balanced with regard to age, body mass, breed, origin, and type of milk feeding (dam, bucket, or automatic feeder) to 2 treatment groups. Animals with identical characteristics were randomly assigned to the groups. In the grass (**GRS**) treatment, the calves grazed on a sward with ryegrass and clover as the main components. In the herb (**HRB**) treatment, ryegrass and clover were supplemented with a greater variety of herb species (18% in total, mainly common chicory, ribwort plantain, and wild carrot). The experimental plots covered a total area of 1.5 ha each. The plots were split into 20 strips of about 0.08 ha. Based on treatment, calves were kept on an assigned strip for 1 to 3 d until a residual height of 4 cm was reached. Sward height was checked using a raising plate meter (True North Technologies Ltd.). The calves had no access to a shed, but the pasture was bordered directly by shady hedges.

The calves were moved to the experimental plots at the age of 108 ± 19 d. Treatment GRS was assigned to 14, 18, and 23 animals in 2018, 2019, and 2020, respectively, whereas HRB included 14, 19, and 23 animals, respectively.

Grazing on the experimental strips lasted 95 ± 17, 62 ± 23, and 77 ± 10 d for GRS, and 94 ± 14, 55 ± 25, and 77 ± 10 d for HRB in 2018, 2019, and 2020, respectively, and excluded grazing with the dam, early grazing, and grazing on adjacent paddocks. Due to episodes of drought causing forage shortages, the animals had to be temporarily relocated to 2 adjacent paddocks (1.5 ha each), both consisting of ryegrass and clover as main components. Grouping of the calves was retained during this period. The average time an individual spent in the adjacent paddock varied between the experimental runs (2018: 30 ± 10, 2019: 48 ± 32, and 2020: 34 ± 16 d·animal^−1^) due to varying weather conditions and also due to different grazing starts depending on the individual age.

Concentrate pellets (500 g·animal^−1^) were provided daily in troughs (35 cm per animal) to maintain the safe handling of the animals. The calves had ad libitum access to water (200-L troughs, Suevia Haiges GmbH) and mineral supplements.

In cases of liquid fecal consistency (score according to Ireland-Perry and Stallings, 1993), hay was additionally provided in hayracks (Patura KG) to stimulate digestion by increasing the amount of crude fiber (Webb et al., 2015). The hay feeding periods were the same in both groups, amounting to 30, 36, and 15 d in 2018, 2019, and 2020, respectively.

The growth and health of the animals were checked weekly according to Roth et al. (2009). Every second week, individual fecal samples were collected to assess the consistency and the level of infestation with intestinal parasites (McMaster flotation with a quantitative microscopic examination; Zajac et al., 2012). If a threshold of 100 eggs·g^−1^ feces (Bystron et al., 2018) was exceeded, the calf was treated with an anthelmintic drug (Panacur Suspension 10%, Intervet Deutschland GmbH).

All 111 calves were clinically healthy at the time of slaughter. Slaughtering took place from June to October at a slaughterhouse 13 km from the research farm. Of each treatment, 4 to 5 calves were delivered at the age of 222 ± 15 d (GRS) and 221 ± 13 d (HRB).

During evisceration of the carcasses, abomasa were severed from the rest of the gastrointestinal tract and cut out as a whole. After removing the abomasal content, every abomasum was packed in a plastic bag, labelled, and kept at a temperature of approximately 2°C until examination. All samples were taken within 2 h after delivery of the calves to the abattoir.

The presence of nonperforating abomasal lesions was assessed visually within 3 h after slaughter following the protocol of Bähler et al. (2010, p. 353). The examiner was neither aware of the animals' identity, as all abomasa were labeled by a second person who was not involved in the study. Macroscopic visual lesions were classified and recorded separately for the fundic and pyloric part. Four types of nonperforating lesions were distinguished, with type 1 denoting the least and type 4 the most severe manifestation. Superficial erosions with minimal defects and discolorations of the mucosa characterized type 1; deeper erosions with a clearly depressed center reflected type 2; type 3 showed craters with a superficial coating, apparent loss of tissue, and central depression; and deep craters with a depressed and hemorrhagic center indicated type 4 (Figure 1). As lesions perforating the entire thickness of the mucosa are often fatal (Palmer and Whitlock, 1984) and thus seldom observed at time of regular slaughter, they were not included in the classification but would have been reported when seen.

**Figure 1:**
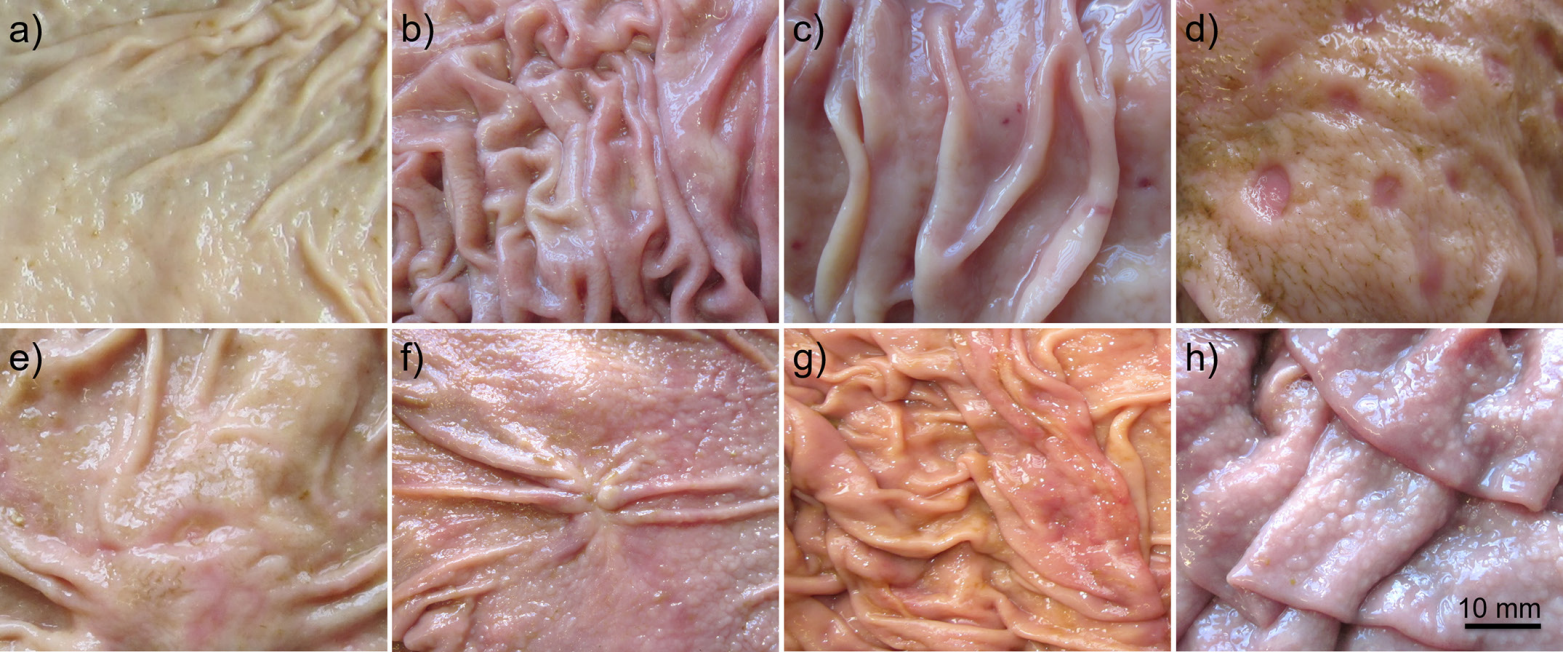
Pathological findings in the fundic and pyloric mucosa of abomasa in dairy bull calves that grazed until an age of 6 to 8 mo. Abomasal lesions categorized according to Bähler et al. (2010, p. 353). Pylorus: (a) free of lesions, (b) diffuse inflammation, (c) type 1: superficial erosions with minimal defects and discolorations, (d) type 2: deeper erosions with a clearly depressed center, (e) type 3 (at *Torus pyloricus*): craters with a superficial coating up to apparent loss of tissue, (f) scar. Fundus: (g) type 2: deeper erosions with a clearly depressed center, (h) parasitic nodules.

Unexpected pathological abnormalities, such as diffuse inflammation, scars (Figure 1f), and parasitic nodules (Figure 1h), were also recorded. Diffuse inflammation appeared in normal mucosal rugae but with slight discoloration that varied in the degree of redness and proliferation of the mucosa (Figure 1b). Inflammatory redness ranged from a mildly coherent reticular pattern to an entirely reddened mucosa, which made it impossible to accurately count the spots.

The scars appeared star-shaped (10 mm in diameter) on the mucosa of the pylorus. Parasitic nodules (1–2 mm in diameter) occurred clustered in the fundus. Another unexpected clinical trait, swollen lymph nodes, was found in adipose tissue surrounding the abomasum. However, these could not be quantified, as some may have been partially severed during slaughter.

Data analyses were performed in R version 4.0.3 (R Core Team, 2020) using generalized linear mixed models (GLMM, package lme4; Bates et al., 2015). Due to the low incidence of unaffected abomasa and lesions of type 3, records were merged into a new outcome variable with 2 categories: category 1 included abomasa without lesions or with type 1 lesions only, and category 2 comprised all abomasa with at least one lesion of type 2 (Figure 2). Treatment (GRS/HRB), breed (German Holstein/Jersey), number of grazing days, and anthelmintic drug use (yes/no) were used as fixed effects. The latter was included because we expected that elimination of parasites would positively affect abomasal health. To determine the potential effect of sward composition, only the number of days on the experimental paddock was considered; days of grazing on adjacent paddocks, early grazing, and grazing with the dam were excluded. Since grazing within treatment occurred on the same plot in each experimental year, treatment nested within year entered the model as a random factor. Since all animals received approximately equal amounts of milk and milk feeding was not part of the main study, these aspects were not considered.

**Figure 2:**
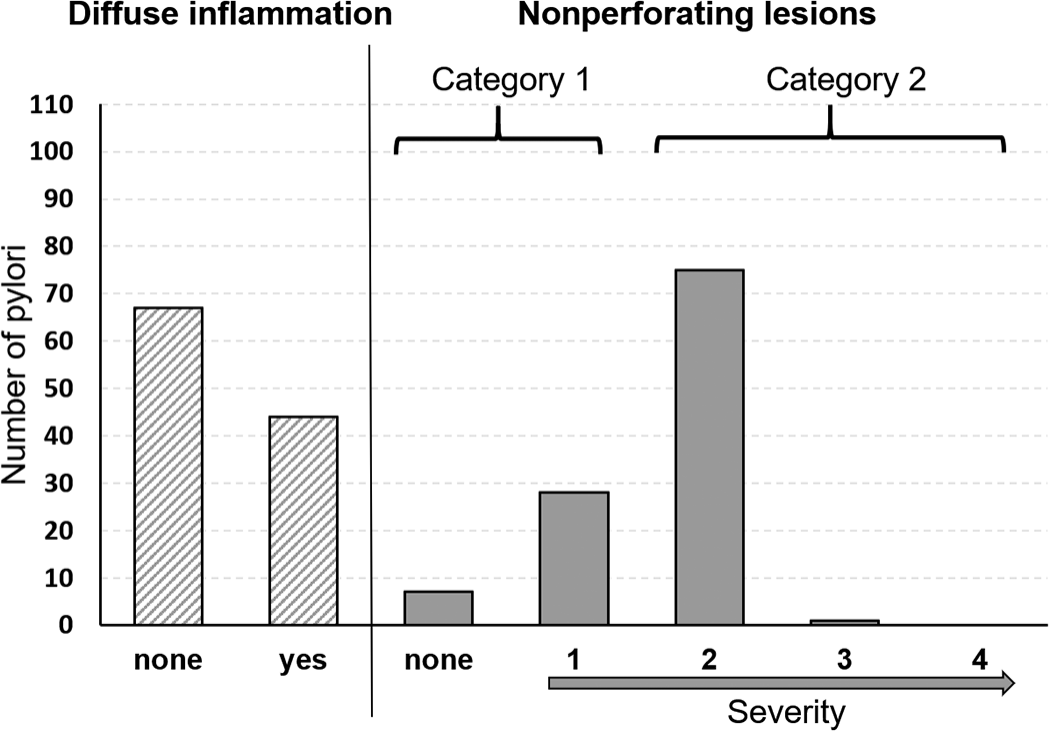
Number of pylori of dairy bull calves (n = 111 in total) affected by diffuse inflammation (yes/none) and of nonperforating lesions (type 1, 2, 3, or 4), classified according to Bähler et al. (2010, p. 353). Category 1 included pylori without lesions or type 1 lesions only, and category 2 comprised all pylori with at least one type 2 lesion.

In addition to the lesions, diffuse inflammation was observed. To determine whether the occurrence of lesions and diffuse inflammation were related, a Pearson's chi-squared test was performed. Since no significant relation was found, the occurrence of diffuse inflammation (yes/no) was set as outcome in a second model that included the same fixed and random predictor variables as before plus the occurrence of lesions (category 1 or 2) as fixed effect. The *P-*values were calculated using a Wald test, and the level of significance was set at *P* < 0.05. The model assumptions were tested using the DHARMa package (Hartig, 2022), and no deviations were detected.

We did not find severe type 4 lesions or lesions perforating the entire thickness of the mucosa.

Seven abomasa were free from lesions, whereas in 104 of the 111 abomasa, nonperforating lesions (types 1 and 2) were found. Lesions of different types occurred separately or simultaneously and were irregularly distributed across the entire mucosa. Type 2 was occasionally associated with the loss of normal mucosal rugae, particularly in the area around the lesion (Figure 1d). In one case, a type 3 lesion (Figure 1e) was found on the *Torus pyloricus* in combination with type 2 lesions. Fundi were less affected than pylori (16 vs. 104).

Over the years, the number of lesions of type 1 and 2 in the pylori varied but without any clear pattern. However, more pylori showed at least one lesion of type 2 than none or only type 1 lesions. Contrary to our hypothesis, abomasal lesions occurred independently of the sward composition (Table 1). One reason could be that the selected herbs have no effect on abomasal lesions, but other species may. However, it is also conceivable that potentially relevant herbs have not become sufficiently established. Furthermore, we could not record the specific herb intake. As cattle are known to graze selectively (Dumont et al., 2007), future studies should monitor the actual intake of herbs and their effect. The unscheduled reduced duration of exposure to the treatments is a strong limiting factor of our study. Nevertheless, 75% of all calves spent more than 50% of their total grazing time on the experimental plots. However, there are no data on how much time is required and how long it takes for a trigger to cause nonperforating lesions. To support our conclusions, further studies are needed in which calves graze according to the treatment (GRS/HRB) for the entire grazing period.

**Table 1:** Model estimates of the tested effects on the incidence risk of nonperforating lesions (category 2 compared with 1) and on the incidence of a diffuse inflammation in the pylorus

Item	Odds ratio	95% CI	*P*-value
Predictor of lesion category (1 vs. 2)			
Intercept	0.49	0.04–5.65	0.56
Treatment (herb vs. grass)	0.77	0.35–2.11	0.74
Breed (Jersey vs. German Holstein)	0.42	0.16–1.06	0.07
Grazing days (experimental paddock)	1.01	0.99–1.03	0.18
Anthelmintic drug use (yes vs. no)	1.74	0.68–4.44	0.25
Predictor of diffuse inflammation (yes vs. no)			
Intercept	0.96	0.13–7.30	0.97
Treatment (herb vs. grass)	1.26	0.55–2.92	0.58
Breed (Jersey vs. German Holstein)	0.52	0.21–1.31	0.17
Grazing days (experimental paddock)	1.00	0.99–1.02	0.78
Anthelmintic drug use (yes vs. no)	0.39	0.17–0.90	0.03
Lesion category (1 vs. 2)	0.93	0.42–2.08	0.86

Diffuse inflammation was recorded in 46 of the 111 abomasa. Thereof, 27 HRB calves and 19 GRS calves were affected. Although 90% of all recorded diffuse inflammation occurred in combination with lesions (44 of 46 abomasa), the presence of lesions (category 1 or 2) in the pylorus was not associated with the occurrence of diffuse inflammation (χ^2^ = 0.7, df = 1, *P* = 0.4).

Swollen abomasal lymph nodes were found in 42 of 111 calves, and a single scar was recorded in the pylorus of 3 abomasa (2019: 1, 2020: 2). Parasitic nodules were found in the fundus once in 2019 and in 7 cases in 2020.

Particularly noteworthy is the absence of severe lesions as reported by Wiepkema et al. (1987). They found nonperforating lesions of type 4 in 23% of 60 pylori of 6-mo-old dairy bull calves that were, in contrast to our study, bucket-fed with milk replacer twice a day, had no access to roughage, and were individually housed in single calf hutches. Munch et al. (2019) reported an even higher prevalence of type 4 lesions in an abattoir survey of Danish Holstein cows: 34% of the 1,326 assessed pylori were affected.

In our study, the most severe manifestation of abomasal damage was one single record of a type 3. Although the overall incidence of lesions was high, their severity was low. This is in contrast to the results of Bähler et al. (2010) who studied veal calves in Switzerland and found type 3 lesions to be most abundant. Reared to an average age of 5 mo, the animals did not have access to an outside pen, were bucket-fed with a liquid milk by-product, and only straw was provided. Under these conditions, 71% of the 61 examined pylori showed some degree of lesions, of which, in turn, 80% were attributed to the more severe type 3 (Bähler et al., 2010). Therefore, our data indicate that pasture-based fattening may not reduce the incidence of abomasal lesions but may reduce their severity. Bähler et al. (2010) showed that chronic stress caused by management and housing can lead to nonperforating lesions in the fundus, whereas lesions in the pylorus are rather linked to dietary aspects. The low number and mild expression of lesions in the fundus suggests that the calves in our study were exposed to less severe continuous stress compared with calves kept without access to pasture. The more frequent occurrence of lesions in the pylorus than in the fundus indicates dietary effects as demonstrated by Brscic et al. (2011), but these authors also pointed out the additional risks due to housing and management.

Grazing allows cattle to express their normal feeding behavior (Charlton and Rutter, 2017). However, the abomasal lesions that occurred in our study raise the question of whether wild ruminants are also affected. Prestwood and Kellogg (1971) reported numerous small ulcers in the abomasum of one 6-mo-old white-tailed male deer (*Odocoileus virginianus*). The authors attributed this to forage competition with free-ranging cattle and wild boars. Although the anatomical differences do not allow a direct comparison between equids and ruminants, the results by Ward et al. (2015) point in a similar direction. They found gastric ulcers in feral horses (12 of 27 affected). Nonetheless, their domesticated counterparts showed a higher prevalence (all 51 affected). Since we found no other studies investigating the occurrence of abomasal changes in free-ranging ruminants or beef calves on pasture, our results require further clarification.

To our knowledge, diffuse inflammation in cattle has not been described previously. Osińska et al. (2010) observed infiltration of inflammatory cells and lesions in the abomasal walls, swollen abomasal lymph nodes, and occasional parasitic nodules in 54 free-ranging European bison. Commonly, these findings were accompanied by intestinal parasites of the genera *Ostertagia* or *Trichostrongylus* that are known to further deteriorate lesions and influence excretory functions of epithelial cells of the abomasum (reviewed by Simpson, 2000). Magdálek et al. (2021) described something similar in 21 abomasa of 5 cervid species. Since some calves in our study were also infected with parasites and both parasitic nodules and swollen lymph nodes were found, it is reasonable to assume that diffuse inflammation was related to their presence. In particular, the lower risk of diffuse inflammation associated with anthelmintic treatment (Table 1) supports this assumption. Since transport to the slaughterhouse increases plasma cortisol levels in veal calves (Van de Water et al., 2003), transport stress may also play a role in formation of the observed diffuse inflammation.

Abomasal scars in 18 of 60 calves were reported by Wiepkema et al. (1987), who interpreted them as remnants of large ulcers. We found such scars in only 3 of 111 calves, and it could be that they suffered from type 4 lesions earlier, possibly even before grazing.

In conclusion, compared with the severe abomasal damage observed in roughage-deprived and space-constrained systems, pasture-based production benefits veal calves' welfare and might be a housing system well accepted by society.
